# Risk of Tuberculosis in Dialysis Patients: A Nationwide Cohort Study

**DOI:** 10.1371/journal.pone.0029563

**Published:** 2011-12-27

**Authors:** Claudia C. Dobler, Stephen P. McDonald, Guy B. Marks

**Affiliations:** 1 Respiratory and Environmental Epidemiology, Woolcock Institute of Medical Research, University of Sydney, Sydney, New South Wales, Australia; 2 Department of Respiratory Medicine, Liverpool Hospital, Sydney, New South Wales, Australia; 3 Austrailia and New Zealand Dialysis and Transplant (ANZDATA) Registry, Adelaide, South Australia, Australia; 4 Discipline of Medicine, University of Adelaide, Adelaide, South Australia, Australia; Fundació Institut d'Investigació en Ciències de la Salut Germans Trias i Pujol. Universitat Autònoma de Barcelona. CIBERES, Spain

## Abstract

**Background:**

The ability to identify individuals at increased risk of developing tuberculosis (TB) has important implications for public health policy and patient care. We conducted a general population historical cohort study in all Australian States and Territories to establish the risk of TB arising in people on chronic hemo- or peritoneal dialysis.

**Methodology/Principal Findings:**

Cases of TB disease in patients receiving chronic dialysis were identified by record linkage using the Australia & New Zealand Dialysis and Transplant Registry (ANZDATA) and State and Territory TB notification databases 2001 to 2006. Main outcome measure was the relative risk of TB in people on dialysis, adjusted for TB incidence in country of birth, sex, age and indigenous status. A total of 6,276 cases of active TB were reported among 19,855,283 people living in Australia between 2001 and 2006. Among 14,506 patients on dialysis, 37 had a notification for TB disease after commencing dialysis, of whom 28 were culture positive. The incidence of TB was 66.8/100,000/year (95% CI 47.7 to 93.2) among people on dialysis and 5.7/100,000/year (95% CI 5.5 to 5.8) in the general population. The adjusted relative risk (aRR) of TB in people on dialysis was 7.8 (95% CI 3.3 to 18.7), and the aRR of culture positive TB was 8.6 (95% CI 3.9 to 19.3).

**Conclusions/Significance:**

Patients on dialysis are at increased risk of TB. The final decision to screen for, and to treat, LTBI in individual dialysis patients will be influenced by a cumulative assessment of the risk of reactivation of TB and by assessment of risk factors for adverse effects of treatment.

## Introduction

Treatment of latent tuberculosis infection (LTBI) in high risk groups such as those who are HIV positive or those in whom anti-TNFα therapy is to be commenced plays an important role in control of tuberculosis (TB) disease in low-TB incidence settings. Patients with chronic renal failure are potentially also a high risk group for TB. Uremia is associated with immunodeficiency caused by functional abnormalities of neutrophils, reduced T and B cell function and impaired monocyte and monocyte-derived dendritic cell function [1], [2], [3], [4], [5]. Additionally, poor nutritional status, vitamin D deficiency and hyperparathyroidism in patients with chronic kidney disease contribute to impaired immunity [6], [7].

An increased risk of TB in dialysis patients was first reported in 1974 [8]. Since then a number of studies have shown that the risk of TB in patients with chronic renal failure and on dialysis is significantly increased [9]. However, most of available data comes from case control studies, many with poorly defined study bases and hence risk of selection bias as well as unmeasured confounding [9]. Population-based cohort studies have described relative risks of TB of 3.4 to 25.3 in dialysis patients compared to the general population [10], [11], [12], [13]. Although selection bias was less of a problem in these population-based cohort studies compared to case-control studies, most made no adjustment, or only very limited adjustment, for confounders (Table 1).

**Table 1:** Population based cohort studies assessing the relative risk of TB in dialysis patients.

Author, year of publication, reference	Location	Background TB incidence 100,0000/year	Number of dialysis patients	Relative Risk(95% CI)	adjusted Relative Risk(95% CI)	potential confounders Relative Risk is adjusted for	stratification by
Ahmed et al, 2004, [10]	California, USA	11.9	2,809	11.3 (*)	not done	none	none
Chou et al, 2001, [11]	Taiwan	71.1	24,553	6.9 (*)	not done	none	gender, age
Simon et al, 1999, [12]	New Jersey, USA	10.7–10.8	4,831	6.9 (*)	3.4 (*)	age, race	none
Chia et al, 1998, [13]	British Columbia, Canada	10.1**	886	*	25.3 (22.9–31.5)	age	none

*information missing.

**for age 20–60 years.

There is currently no uniform approach to screening of patients with chronic renal failure who are on dialysis. Some international guidelines advocate screening patients on dialysis for LTBI [14], others do not recommend routine assessment for LTBI in this patient group [15]. In order to inform health policy decisions about screening for LTBI in potential high risk groups, such as people on dialysis, robust estimates of the risk of TB are required. We conducted a nation-wide, general population historical cohort study to estimate the risk of TB among people on hemo- or peritoneal dialysis with adjustment for several important, potentially-confounding, risk factors.

## Methods

### Ethics Statement

The study protocol was approved by the Sydney South West Area Health Service Human Research Ethics Committee - Western Zone, the New South Wales Population & Health Services Research Ethics Committee, the Australian Institute of Health and Welfare Ethics Committee, the Queensland Health Research Ethics & Governance Unit, the Department of Human Services Victoria Research Governance, the Australian Capital Territory Health Human Research Ethics Committee, the Department of Health Western Australia Human Research Ethics Committee, the Tasmania Health and Medical Human Research Ethics Committee, the South Australia Department of Health Human Research Ethics Committee and the Northern Territory Human Research Ethics Committee.

The requirement for written or verbal patients' consent for this data linkage study was waived by all of the above mentioned ethics committees based on a combination of the following criteria: The size of the population, the proportion of individuals who are likely to have moved or died since the health information was originally collected, the risk of introducing potential bias into research and thereby affecting the generalisability and validity of the results, the risk of creating additional threats to privacy by having to link information in order to locate and contact individuals to seek their consent, the difficulty of contacting individuals directly when there is no exisiting or continual relationship between the organisation and the individual and the difficulty of contacting individuals indirectly through public means, such as advertisments or notices. Only de-identified information was made available to the researchers.

### Setting and study population

The study cohort was all 19.9 million Australian residents in 2006, 14,506 of whom were on chronic hemo- or peritoneal dialysis (non-transplant recipients). Australia has a low incidence of TB of 5.4 per 100,000 population, and over 80% of all TB cases occur in overseas-born people [16].

### Description of data sources and data linkage

#### Australian and New Zealand Dialysis and Transplant Registry

People on dialysis were identified using the Australian and New Zealand Dialysis and Transplant (ANZDATA) Registry. ANZDATA collects information from all renal units in Australia and New Zealand. Information collected includes, but is not limited to, primary renal disease, comorbidities, type of dialysis, transplant date, drug dosages and country of birth. The full data collection form as well as detailed information on the ANZDATA registry have been published online [17].

#### State and Territory TB notification databases

All TB treatment is provided free-of-charge of State and Territory health authorities, who also undertake public health action in relation to each case. Notification of TB to these health authorities is compulsory as this notification initiates the public health investigation and action.

We used the State and Territory TB notification databases to identify patients with TB. All individuals who were notified as a case of TB disease between January 2001 and December 2006 were included in our analyses. Data on names, date of notification, date of birth, sex, country of birth, indigenous status and TB culture results were extracted for this analysis. Diagnosis of TB in culture negative cases was based on the finding of clinical, radiological and histological features typical for pulmonary or extra-pulmonary TB.

#### Data linkage

People on dialysis who had an episode of active TB were identified by record linkage using the ANZDATA and the State and Territory TB notification databases from January 2001 to December 2006. Probabilistic linkage was performed by the Cancer and Screening Unit of the Australian Institute of Health and Welfare (AIHW). They used names as the primary linkage key with sex and date of birth used for confirmation or checking. Match files were created containing only a unique record identifier for both datasets and the record linkage identifier. From each of the original datasets, analysis files were created containing the unique record identifier and all the data fields required for analysis. Each of the analysis files was then linked to the match files and the record identifiers were removed from these analysis files. Hence, anonymized linked files were sent to us for analysis.

#### Census data for the general population

Aggregate Census population data for 2006 classified by strata of age group, country of birth, sex, and indigenous status were obtained from the Australian Bureau of Statistics. Age was grouped into 5 year age groups and country of birth was aggregated to groupings of countries with a similar incidence of TB (<10, 10–24, 25–49, 50–99, 100–299, and ≥300 per 100,000) based on published World Health Organization data [18].

The term indigenous is used to refer to Australian Aboriginal and Torres Strait Islander peoples. It is based on responses to an Australian Bureau of Statistics standard question for Indigenous identification, which is used in self-enumerated collections as the Census 2006. [19].

### Sample size and study power

The annual incidence of TB in Australia is 5.4 per 100,000 [16]. Hence, over six years, the expected cumulative incidence is 32 per 100,000. The study population is the entire population of Australia, that is, approximately 20 million people. We estimated that there were 13,000 persons on dialysis. The study had 80% power to detect a five-year cumulative incidence >84 per 100,000 in people on dialysis. This represents a detectable relative risk of 2.63 or higher which is substantially lower than the 10- to 15-fold expected increase in risk.

### Statistical analysis

The relative risk of TB in patients with DM was estimated using a log-binomial model with correction for overdispersion and adjustment for TB incidence in country of birth, sex, age and indigenous status. Aggregate population data for these covariates were obtained from the Census as described above. The population attributable fraction was estimated using the formula: (Pe * (RR−1))/((Pe * (RR−1)+1) where RR is the relative risk, estimated as above, and Pe is the proportion of the population exposed to the risk factor, that is, the prevalence of chronic dialysis-treated patients in the population [20]. Individual level data on potential confounders were available for the dialysis and TB cohorts. For the general population, we used aggregated data on the prevalence of potential confounders derived from the census. For this purpose, we obtained a contingency table from the Australian Bureau of Statistics containing population numbers cross-classified by all possible combinations of strata of the covariates listed above. Only cases of TB that were notified after the commencement of dialysis were included. The follow-up period started from 1/1/2001 or the date of commencement of dialysis, whichever was the later and continued until 31/12/2006 or the date of diagnosis of TB, whichever was the earlier. TB incidence rates were expressed per 100,000 person years of follow up with asymptotic 95% confidence intervals [21].

All statistical analyses were carried out using SAS Statistical Software (Version9.2) (SAS Institute, Cary, NC, USA).

## Results

### Description of the cohort

During the study period 14,506 patients received dialysis (non-transplant recipients); the general population comprised 19,855,283 residents of Australia. Characteristics of the dialysis population, the general population, the TB population and the dialysis population with TB are shown in table 2. The proportion of Australian-born people was lower in the dialysis population compared to the general population. The proportion of dialysis patients who were born in a country with a TB incidence ≥25/100,000 was higher than in the general population. Indigenous people were over-represented among patients on dialysis compared to the general population; 11% of all patients on dialysis were indigenous whereas only 2% of the general population were indigenous.

**Table 2:** Characteristics of the general population, the dialysis population, the TB population and the TB in dialysis population.

Variable	General population including people on dialysis (n = 19,855,283)	Dialysis population (n = 14,506)	TB population (n = 6,276)	TB in dialysis population, limited to TB cases after start of dialysis(n = 37)
**Gender, number (%)**				
Female	10,056,036 (51)	6,158 (42)	3045 (49)	21 (57)
Male	9,799,247 (49)	8,348 (58)	3231 (51)	16 (43)
**Age group in years, number (%)**				
<15	3,937,213 (20)	33 (0)	272 (4)	0
15–34	5,380,655 (27)	440 (3)	2220 (35)	2 (5.4)
35–54	5,700,354 (29)	2,533 (17)	1729 (28)	2 (5.4)
55–74	3,566,112 (18)	6,638 (46)	1176 (19)	21 (56.8)
≥75	1,270,949 (6)	4,862 (34)	879 (14)	12(32.4)
**Country of origin, number (%)**				
Australian born	14,072,943 (71)	9,677 (67)	1212 (19)	6 (16)
born overseas	4,416,033 (22)	4,829 (33)	5064 (81)	31 (84)
Unknown	1,366,307 (7)	0	0	0
**Indigenous status, number (%)**				
non-indigenous or not-stated	19,402,435 (98)	12,939 (89)	6072 (97)	32 (86)
Indigenous	452,848 (2)	1,567 (11)	204 (3)	5 (14)
**TB incidence in country of birth (per 100,000), No. (%)**				
<25	16,792,180 (85)	12,933 (89)	1964 (31)	9 (24)
25–99	884,709 (4)	905 (6)	1350 (22)	11 (30)
≥100	754,491 (4)	668 (5)	2962 (47)	17 (46)
Unknown	1,423,903 (7)	0	0	0

### Incidence of TB

During the study period, there were 6,276 TB notifications in the whole Australian population of whom 4,423 (70% of all TB notifications) were culture positive (Table 2). The overall incidence of TB in the general population over this period was 5.7 per 100,000 per year (95% confidence interval 5.5 to 5.8) and the incidence of culture positive TB was 4.0 per 100,000 per year (95% CI 3.9 to 4.1) (Table 3). Among 14,506 patients on dialysis there were 37 cases of TB that were registered after the commencement of dialysis. Of these, 28 (76%) were culture positive. Five (14%) occurred in indigenous people. The mean duration of follow-up was 3.8 years. The overall incidence of TB among patients on dialysis was 66.8 per 100,000 person-years (95% CI 47.7 to 93.2) (Table 3) and the incidence of culture positive TB was 50.6 (95% CI 34.3 to 74.2) per 100,000 person-years.

**Table 3:** Incidence of tuberculosis in the general population and in people on dialysis.

	Incidence of all TB per 100,000/year(95% confidence interval)		Incidence of culture positive TBper 100,000/year(95% confidence interval)	
	General population	People on dialysis	General population	People on dialysis
**All persons**	5.7 (5.5 to 5.8)	66.8 (47.7 to 93.2)	4.0 (3.9 to 4.1)	50.6 (34.3 to 74.2)
**Age, years**				
<15	1.2 (1.1 to 1.4)	0	0.4 (0.3 to 0.5)	0
15 to 34	7.4 (7.1 to 7.7)	163.3 (28.3 to 656.2)	5.5 (5.2 to 5.8)	81.6 (4.3 to 528.5)
35 to 54	5.5 (5.2 to 5.7)	24.3 (4.2 to 98.1)	3.9 (3.7 to 4.1)	12.2 (0.6 to 79.0)
55 to 74	6.0 (5.6 to 6.3)	88.5 (56.2 to 137.8)	4.1 (3.8 to 4.4)	67.4 (39.9 to 112.1)
≥75	13.0 (12.1 to 13.9)	59.9 (32.5 to 107.8)	9.7 (9.0 to 10.5)	49.9 (25.4 to 95.1)
**Sex**				
Male	5.9 (5.7 to 6.2)	51.0 (30.2 to 84.9)	4.3 (4.1 to 4.5)	35.1 (18.5 to 64.9)
Female	5.4 (5.2 to 5.6)	87.5 (55.6 to 136.2)	3.7 (3.6 to 4.9)	70.8 (42.6 to 116.0)
**TB incidence in country of birth**				
<25 per 100,000	1.95 (1.86 to 2.04)	18.2 (8.9 to 36.0)	1.26 (1.20 to 1.34)	14.2 (6.2 to 30.6)
25–99 per 100,000	25.5 (24.1 to 26.9)	315 (166 to 582)	18.0 (16.9 to 19.2)	258 (126 to 508)
≥100 per 100,000	65.4 (63.1 to 67.8)	698 (420 to 1140)	48.8 (46.8 to 50.9)	493 (267 to 885)

### Relative risk of TB

The unadjusted relative risk (RR) of TB in people on dialysis was 17.1 (95% CI 5.7 to 51.4) for all cases and 19.9 (95% CI 7.0 to 56.4) for culture positive cases. In the multivariate analysis we adjusted for age, TB incidence in country of origin, indigenous status and sex. The adjusted relative risk (aRR) of TB in people on dialysis was 7.8 (95% CI 3.3 to 18.7) for all cases and 8.6 (95% CI 3.9 to 19.3) for culture positive cases, respectively.

The population attributable fraction was 0. 3% based on a dialysis prevalence of 44.7 per 100,000 population in 2006 [17].

## Discussion

In this large, population-based cohort study conducted in 19.9 million residents of Australia we found that people on dialysis have a seven to eight-fold increased risk of developing TB compared with the general population.

The relative risk of TB among patients on dialysis in our study, although significantly increased, is at the lower end of the range of results reported from many of the previously published case-control studies and comparable to the findings in three out of the four published population-based cohort studies (Table 1). A review by Hussein et al. listed 28 studies on TB in dialysis patients which had relative risks ranging from 6.9 to 52.5 [9]. In population based cohort studies (Table 1) relative risks were 11.3 and 6.9, respectively, in two studies that did not adjust for potential confounders [10], [11] and 3.4 and 25.3, respectively, in two studies that did adjust for some confounding factors [12], [13]. Of the previously published population-based studies, only a US study from New Jersey adjusted for age and race. In that study, the adjusted relative risk was 3.4 [12]. In a Canadian study the incidence of TB in dialysis patients was 25.3 times higher than the incidence in the general population aged 20 to 60 years [13]. This restricted age for the reference population was chosen to be similar to the age range of the dialysis population. However, the estimated relative risk was not adjusted for TB incidence in country of birth [13]. As TB incidence in country of birth is an important confounder for studies of TB that are conducted in low incidence settings, it is possible that confounding by differences in TB incidence in country of birth, led to the high relative risk estimate of 25.3 in the Canadian study.

The incidence of TB increases with age in low incidence settings, and people on dialysis tend to be older than the general population. Hence, adjustment for age, as a potential confounder is important. It has been previously demonstrated in the ANZDATA population that foreign born people are over-represented among dialysis patients [22]. This is likely to reflect differences in the prevalence of diabetes and various forms of glomerulonephritis among the foreign-born population compared to the Australian-born population. This study has shown that the incidence of TB in the country of birth is one of the strongest predictors of the risk of developing TB. This is because people born in countries where TB is endemic are more likely to have LTBI and remain at risk of developing (reactivation) TB. Thus, not adjusting for country of birth will result in overestimation of the relative risk of TB among people on dialysis. This adjustment is especially important for low TB incidence settings, where the majority of TB cases usually occur in foreign-born people [23].

HIV is a well known risk factor for TB but the prevalence of HIV infection is low in Australia [24] and HIV-TB co-infection is probably uncommon [25]. However, it is acknowledged that HIV status was reported for only 35% of all Australian TB cases in 2006 [25]. The relatively low prevalence of HIV infection among migrants from countries with a high domestic prevalence of HIV infection is probably explained by the “healthy migrant effect” [26]. Intending migrants are screened for HIV infection during the visa application process. Dialysis patients are routinely screened for HIV, but this information is not systematically collected in ANZDATA. It is possible that a higher prevalence of HIV infection among the dialysis cohort could have contributed to the increased TB risk. However, rates of HIV nephropathy (reported as the primary cause of end-stage kidney failure) are extremely low in Australia [17]. HIV infection thus is an unlikely to be an important confounder in the between dialysis status and TB disease in Australia.

Our study included patients on hemodialysis as well as patients on peritoneal dialysis. Patients on hemodialysis and peritoneal dialysis share the same risks associated with acquired immunodeficiency due to uraemia. A Chinese study that examined the risk of TB in 790 patients undergoing continuous ambulatory peritoneal dialysis found a RR of 7 in this group compared to the general population [27]. This result would be in keeping with the range of RRs reported in studies of patients on hemodialysis.

Major strengths of our study are the large size and the general population-based nature of the cohort. This has resulted in estimates that are both reliable and generalisable. A limitation that this study shares with previously published papers in this area is that we did not have any information on treatment of LTBI in the dialysis cohort. Routine assessment and treatment for LTBI in dialysis patients is currently not recommended in Australia, and it can therefore be assumed that most patients on dialysis would not have been assessed and treated for LTBI. However, it is possible that some renal physicians and renal units would have screened and treated dialysis patients for LTBI, thus lowering the risk in this cohort of developing active TB. While screening of dialysis patients for latent TB infection is not standard practice in Australia, patients with chronic renal failure awaiting renal transplantation are commonly screened and treated for LTBI before going on a renal transplant waiting list. For this reason we did not include data on renal transplant patients that were also available from ANZDATA in this study, as the true risk of developing TB likely would have been underestimated.

Other conditions known to be associated with an increased risk of TB include TNFalpha-blocker therapy [28] and being a close contact of a patient with active TB [29]. People in these groups, along with HIV positive individuals, are routinely screened for LTBI in low TB-incidence settings. We have shown here that dialysis patients do have a substantially increased risk of TB compared to the general population, although the increase is not as great as for HIV positive status, TNFalpha blocker therapy or a history of recent TB contact. While our study provides important information to inform the public health and clinical decision making process about LTBI screening in patients on dialysis, it was not designed to evaluate the overall benefit of LTBI screening among dialysis patients. Further studies are required to examine the effectiveness and cost-effectiveness of such an intervention.

This study confirms that the risk of TB in dialysis patients is substantially increased. The absolute risk of TB in dialysis patients without other risk factors for TB may not be sufficiently high to warrant screening for LTBI. However, in dialysis patients with additional risk factors, especially in those who were born in TB endemic countries, screening for, and treatment of, LTBI should be considered. Further studies are required to investigate the cost-effectiveness of such an intervention. The final decision to treat LTBI in individual patients will be influenced, on the one hand, by factors associated with risk of adverse effects of treatment, such as age and co-morbid liver disease, and on the other hand by additional factors affecting the cumulative risk of reactivation of TB including life-expectancy and the presence of other co-morbid conditions.
